# Hydroxytyrosol as a Promising Ally in the Treatment of Fibromyalgia

**DOI:** 10.3390/nu12082386

**Published:** 2020-08-09

**Authors:** Jorge A. Ramírez-Tejero, Esther Martínez-Lara, M Ángeles Peinado, María Luisa del Moral, Eva Siles

**Affiliations:** Department of Experimental Biology, University of Jaén, 23071 Jaén, Spain; jrtejero@ujaen.es (J.A.R.-T.); elara@ujaen.es (E.M.-L.); mapeinado@ujaen.es (M.Á.P.)

**Keywords:** fibromyalgia, hydroxytyrosol, extracellular matrix, energy metabolism

## Abstract

Fibromyalgia (FM) is a chronic and highly disabling syndrome, which is still underdiagnosed, with controversial treatment. Although its aetiology is unknown, a number of studies have pointed to the involvement of altered mitochondrial metabolism, increased oxidative stress and inflammation. The intake of extra virgin olive oil, and particularly of one of its phenolic compounds, hydroxytyrosol (HT), has proven to be protective in terms of redox homeostatic balance and the reduction of inflammation. In this context, using a proteomic approach with nanoscale liquid chromatography coupled to tandem mass spectrometry, the present study analysed: (i) Changes in the proteome of dermal fibroblasts from a patient with FM versus a healthy control, and (ii) the effect of the treatment with a nutritional relevant dose of HT. Our results unveiled that fibroblast from FM show a differential expression in proteins involved in the turnover of extracellular matrix and oxidative metabolism that could explain the inflammatory status of these patients. Moreover, a number of these proteins results normalized by the treatment with HT. In conclusion, our results support that an HT-enriched diet could be highly beneficial in the management of FM.

## 1. Introduction

Fibromyalgia (FM) is a chronic syndrome whose main symptom is the widespread pain caused by both allodynia and hyperalgesia [1]. Nonetheless, the FM clinic includes a myriad of physical disturbances like joint stiffness, muscular twitches, burning skin, headaches or gastrointestinal alterations, as well as several psychological disorders and cognitive difficulties [2]. Consequently, this syndrome can be considered as a highly disabling condition that affects to 0.2–6.6% of the general population [3], with an estimated healthcare cost over 5000€ per patient every year [4]. Nowadays, neither a healing treatment nor an accurate biomarker-based diagnostic have been unveiled. As a result, the efforts of the scientific community are still focused on depicting the molecular disturbances that might be contributing to the appearance and chronification of such a wide range of symptoms.

There are many factors related to the etiopathogenesis of FM. It has been proposed that central sensitization has a key role in the chronic pain suffered by these patients. This sensitization could be mainly linked to an abnormal performance of ascending and descending neurological pathways involved in pain processing [1,5,6]. Additionally, it has been observed that peripheral silent C nociceptors have a marked hyperexcitability that could be related with aberrant pain perception in FM patients [7]. Apart from neurological causes, there are a number of molecular disturbances that have been studied in different models. In this context, the role of oxidative stress has been recursively highlighted by scientific literature. In FM patients, there is an increased oxidative stress [8] and a compromised antioxidant defence in terms of low levels of reducing compounds such as ceruloplasmin or copper, and a lower activity of antioxidant enzymes such as superoxide dismutase, glutathione peroxidase and catalase [9]. Additional studies have also pointed to the involvement of oxidative stress in FM, since an impaired mitochondrial metabolism in leukocytes and fibroblasts from FM patients has been found [10,11,12]. Oxidative stress is highly related to inflammation. In FM, inflammation is described as a ‘neurogenic’ [13] or as a ‘low-grade’ based systemic symptom [14]. Both circumstances have been confirmed in cerebrospinal fluid and plasma from patients by an inflammation-related protein panel [15], pointing to protein-related techniques as key partners in this complex syndrome. However, it has not been fully addressed yet how these conditions contribute to the appearance and development of FM symptoms.

Although there is a plethora of drugs currently used for FM treatment, none of them seem to be completely effective by itself. As a result, a combination of pharmacological and nonpharmacological treatments seems to be the more usual approach [16]. Even more, treatments that do not use drugs at all are strongly recommended by several organisms and researchers [17,18]. In line with this hypothesis, nutritional interventions and supplementations have already been used in the management of FM in order to improve the quality of life of these patients. Given that FM has been related to a deficiency in essential metal ions and vitamins, the regular intake of antioxidant compounds could be beneficial to a certain extent [19]. Therefore, balanced diets with high levels of antioxidant components, such as the Mediterranean Diet, could have a potential restoration effect in FM. In this nutritional pattern, the main source of fat is Extra Virgin Olive Oil (EVOO), a vegetable oil with an overwhelming list of proven benefits on human health [20]. In fact, EVOO consumption in FM patients improved their quality of life and health self-perception, as well as decreased oxidative stress markers, such as protein carbonylation and lipid peroxidation [21]. The main actors in the anti-inflammatory and antioxidative effects of EVOO are its phenolic compounds, especially hydroxytyrosol (HT) [22]. This amphipathic phenol is mainly originated by the hydrolysis of secoiridoids during fruit ripening [23] and its concentration in olive oil differs markedly depending on the cultivar, being especially high in ‘Picual’ (up to 600 mg/kg) [24]. The nutraceutical properties of HT have been demonstrated in a wide range of pathologies like cancer, cardiovascular diseases and diabetes [24], most of them linked to its outstanding anti-inflammatory and antioxidant effects [25,26,27].

Since FM is a multifaceted syndrome, the use of omics techniques in its research could be extremely advantageous. In fact, some studies applying genomics [28], transcriptomics [29] and proteomics [30,31,32] published in the last decade have been crucial to unravel a huge number of genes and molecular pathways involved in FM.

Given the reported inflammation and oxidative imbalance in FM patients, as well as the nutraceutical properties showed by HT, in this study we evaluated whether this phenol could be an effective ally in the management of FM. For that purpose, and using a proteomic approach in primary fibroblast cultures, we began by elucidating FM-associated molecular changes in order to analyse whether a nutritional relevant dose of HT can ameliorate them.

## 2. Materials and Methods

### 2.1. Fibroblasts Cultures

Two primary dermal fibroblasts cultures from middle-aged Caucasian women were used in this study: One from a 45-year-old FM patient (Body Mass Index = 26.2) diagnosed by the American College of Rheumatology criteria (FM) and one from a 43-year-old healthy (C) volunteer (Body Mass Index = 25.7). Both women had neither biochemical nor diagnostic imaging abnormalities, and both cultures have been used and described in a previous study [33]. Cells were cultured in DMEM-high glucose (4.5 mg/L) medium (Lonza; Basel, Switzerland), supplemented with 20% fetal bovine serum (Thermo Fischer-Scientific; Waltham, MA, USA), 100 U/L of penicillin, 0.1 µg/mL of streptomycin and 0.25 µg/mL B of amphotericin (Merck; Darmstadt, Germany) at 37 °C, 5% CO_2_.

### 2.2. Hydroxytyrosol Treatment

Fibroblasts were treated during 48 h with HT 1.5 µM (purity ≥ 98%; Extrasynthese; Genay, France), a treatment previously used noncytotoxic concentration [34,35,36], dissolved in pure ethanol (ACS grade) and freshly prepared. Control cells were similarly treated with the same quantity of ethanol.

### 2.3. Proteomic Study

#### 2.3.1. Preparation of Protein Samples and nanoLC-MS/MS Analysis

Cells from each experimental condition were collected by trypsinization and centrifuged at 1500 g. After two washes with PBS, the cell pellets were lysed with a 4% Chaps buffer containing urea (7 M), thiourea (2 M) and DTT (200 mM). Protein extracts were then tryptically digested following, with minor variations, the filter-aided sample preparation (FASP) protocol described by Wisniewski et al. [37]. The resulting peptides were dried, resuspended in 0.1% formic acid and submitted to sonication for 5 min as a preparatory step to nano-liquid chromatography coupled to tandem mass spectrometry (nLC-MS/MS) analysis. Peptide separation was carried out on a nanoACQUITY UPLC System (Waters; Milford, MA, USA) connected to an LTQ Orbitrap XL mass spectrometer (Thermo Electron, Thermo Fischer-Scientific; Waltham, MA, USA). Samples were loaded onto a Symmetry 300 C18 UPLC Trap column (180 μm × 20 mm, 5 μm; Waters), using a precolumn connected to a BEH130 C18 column, 75 μm × 200 mm, 1.7 μm (Waters; Milford, MA, USA) and equilibrated in 3% acetonitrile and 0.1% formic acid. After liquid chromatography step, the eluted peptides were directly placed into the nanoelectrospray capillary (Proxeon Biosystems; Odense, Denmark) at 300 nL/min, applying a 120-min linear gradient of 3–50% acetonitrile. The mass spectrometer automatically switched between MS and MS/MS acquisition in data-dependent acquisition mode (DDA), in an alternating fashion. Full MS survey spectra (*m*/*z* 400–2000) were acquired in the Orbitrap with 30,000 resolution at *m*/*z* 400, and two lock-masses (445.120024 and 462.146573) were set for increased accuracy. The six most intense ions were subjected to collision-induced dissociation (CID) in the linear ion trap, and precursors with charge states of 2 and 3 were selected for fragmentation. To cover a higher range of peptides, analysed ions were excluded from further analysis during 30 s through dynamic exclusion lists. Database searches were performed using the software Proteome Discoverer v.1.4 (Thermo Fischer-Scientific; Waltham, MA, USA).

#### 2.3.2. Differential Expression

To study protein variation among the samples, the resulting proteomic spectra were analysed with Progenesis LC-MS software (Nonlinear Dynamics Ltd.; Newcastle upon Tyne, UK). Raw files were loaded onto the program using the workflow provided by the manufacturers. For relative quantitation, one of the samples was selected as a reference to which the precursor masses in all the other samples’ runs were aligned. Thus, relative abundance ratios between the reference and the query runs were calculated for all features at given retention times. Mascot search engine v2.1 software (Matrix Science; Boston, MA, USA) was used to perform protein database searches. Carbamidomethylation of cysteines was set as fixed modification, oxidation of methionines was set as variable modification, and two missed tryptic cleavages were allowed. Furthermore, 10 ppm of peptide mass tolerance and a fragment mass tolerance of 0.5 Da were used. All samples’ spectra were compared against Uniprot/Swissprot database version 2016_02 restricted to *Homo sapiens*. A decoy search was carried out in order to estimate the false discovery rate (FDR), establishing the threshold at < 1%. Protein relative quantitation was performed according to the intensity of the three most intense peptides (when available), and only proteins with *p* < 0.05 (by ANOVA), a fold change (FC) < 0.8 or >1.2, and at least two peptides accomplishing FDR filter were considered as significantly deregulated. Furthermore, to determine the proteins that were normalized in FM cultures after HT treatment, only those proteins with a FC < 0.5 or >1.5 when comparing FM and C cultures were considered, in order to focus the study in those molecules with a higher response to the treatment.

### 2.4. Bioinformatic Analysis

The relation between differentially expressed proteins and physiological and pathological processes was studied using *Canonical Pathways*, *Network-building* and *Tox terms* tools from Ingenuity Pathways Analysis (IPA; Ingenuity^®^ Systems, www.ingenuity.com). PANTHER database [38] was also used to perform an explorative Gene Ontology (GO) analysis when necessary, comparing the protein list with the PANTHER GO-Slim Biological Processes database. Additionally, KEGG database [39] was used to better characterize the role of these proteins from a different point of view.

### 2.5. Validation of Relevant Proteins

The differential expression found by nLC-MS/MS in certain proteins with relevant roles in crucial pathways was validated by additional techniques. Hence, the expression of collagen type 1 and 6 alpha-1 chains (COL1A1, COL6A1) was determined by western-blot. Briefly, 15 µg of denatured total-protein were separated by electrophoresis as described by Laemmli [40] on a 7.5% polyacrylamide gel. Then, proteins were transferred to a vinylidene polyfluoride membrane (Merck; Darmstadt, Germany) and blocked with blocking buffer (3.5% *W/V* skimmed milk powder in 25 mM of Tris-HCl pH 7.6, 137 mM of NaCl, 2.6 mM of KCl and 0.1% Tween-20). Monoclonal primary antibodies (COL1A1: sc-293182; COL6A1: cs-377143, Santa Cruz Biotechnology; Dallas, TX, USA) were added in blocking buffer. After primary antibody incubation, membranes were washed and incubated with the secondary antibody (1/5000, anti-mouse, A0168, Merck; Darmstadt, Germany). The antigen-antibody complex was detected by chemiluminescence using the commercial ECL Plus kit (GE Healthcare; Chicago, IL, USA). As references for loading control, immunodetection of α-Tubulin was performed with a monoclonal antibody (T5168, Merck; Darmstadt, Germany). Densitometric quantification of the autoradiographs was carried out with the TotalLab program v1.11 (TotalLab Ltd.; Newcastle upon Tyne, UK). Additionally, ELISA tests were used to determine fibronectin 1 (FN1; Cusabio; Houston, TX, USA), catenin beta 1 (CTNNB1) and cofilin 1 (CFL1) (both from Cloud-Clone Corp.; Katy, TX, USA), following manufacturers’ recommendation.

### 2.6. Statistical Analysis

Data were expressed as the mean ± SD of at least three independent values. The absence of differences between compared groups was considered as the null hypothesis (H_0_), establishing the statistical significance to null hypothesis rejection at 0.05 (type I error, α = 0.05). To determine data distribution, Kolmogorov-Smirnov test was applied before each comparative analysis. If data were normally distributed, the difference between the means of the compared groups was made using the Student’s *t*-test for unpaired measurements. Conversely, if the data did not follow a normal distribution, these differences were assessed with the non-parametric Mann-Whitney U test. In the case of nominal categorical variables comparison for GO terms analysis, the hypotheses were tested by Fischer Exact test. Statistical analysis were performed in GraphPad Prism^®^ 6.01 (GraphPad Software Inc.; CA, USA) and R statistical software [41], using *ggplot2* package [42] to build charts.

## 3. Results

### 3.1. Proteomic Signature of FM

As starting point, we compared the proteomes of fibroblasts from the healthy volunteer and from the FM patient. Among the 816 proteins detected, 357 (43.75%) were differentially expressed (Table S1) in the cells from the FM patient (FDR < 1%; *p* < 0.05), 154 overexpressed (FC > 1.2) and 203 underexpressed (FC < 1.2).

#### 3.1.1. GO Analysis

Since the number of differentially expressed proteins in the cell proteome of fibroblasts from the patient was large, a preliminary GO exploratory analysis with PANTHER was conducted. Sorted by fold enrichment, the top 20 statistically significant GO terms are listed in Table 1.

According to PANTHER database, most of the Biological Processes linked to FM were related to the development and maintenance of the muscle-skeletal system and to relevant cellular processes such as translation or protein assembly.

#### 3.1.2. IPA Analysis

**Figure 1:**
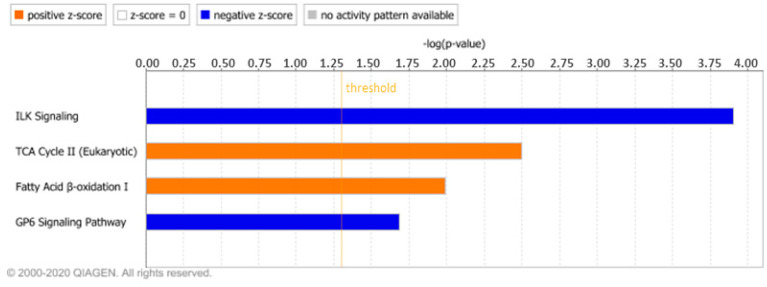
Canonical pathways enriched in fibromyalgia sorted by statistical significance. Orange and blue mean positive or negative Z-score, respectively. Orange line set the threshold established for *p* value (log (0.05) = 1.30).

To further unveil the physiological and pathological processes related with FM, we also analysed the differentially expressed proteins in the fibroblasts of the patient through the IPA software. The Canonical Pathways tool showed four terms that had a significant z-score (|z| > 2; Figure 1), of which two were activated (‘TCA Cycle’ and ‘Fatty acid β-oxidation’) and two were inhibited (‘ILK signalling’ and ‘GP6 signalling pathway’) according to this value. Activated pathways were linked to mitochondrial metabolism, while inhibited ones were related with membrane receptors signalling.

**Figure 2:**
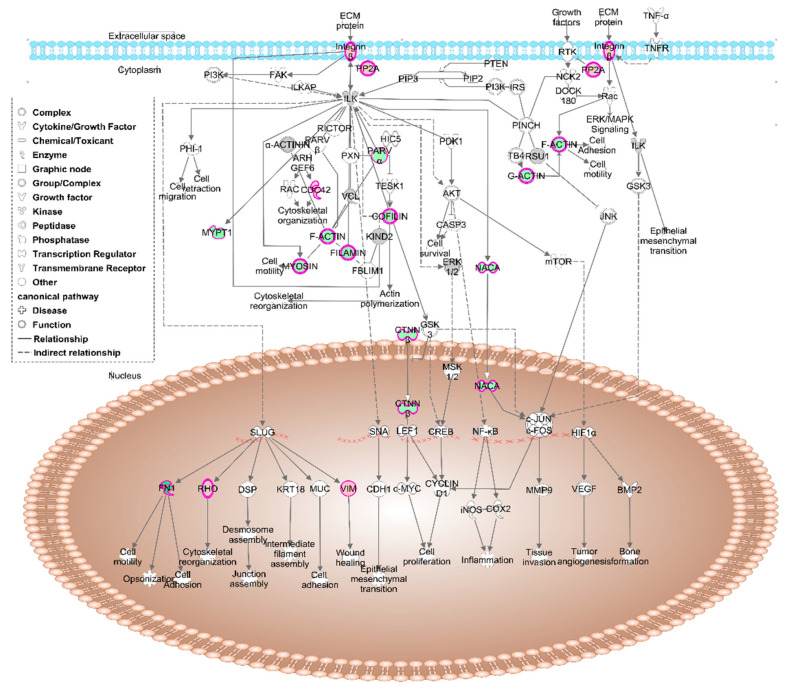
Schematic representation in IPA of the ILK signalling pathway. Red indicates the overexpressed proteins in fibromyalgia (FM) fibroblasts and green indicates the underexpressed ones.

The process with the highest number of indexed molecules was ‘ILK signalling’ (Figure 2), with sixteen proteins annotated (Table 2).

**Figure 3:**
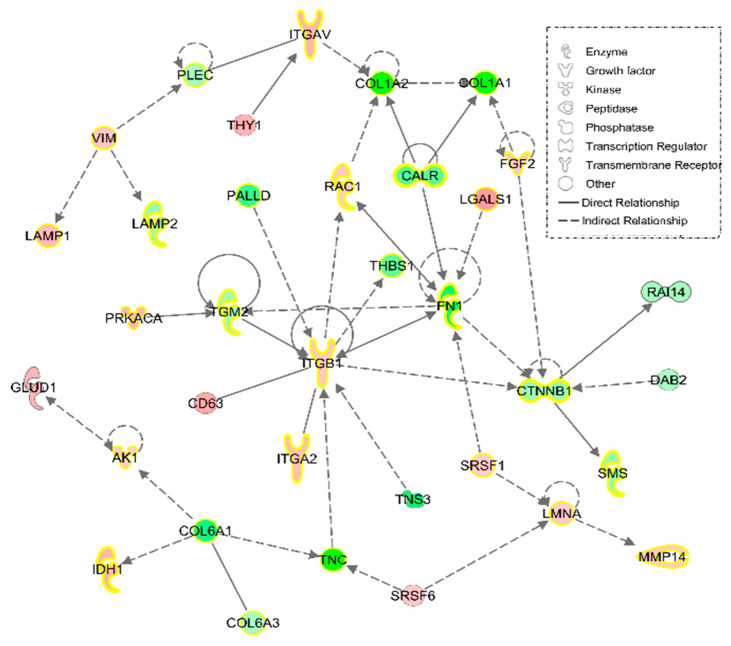
‘Cell morphology, cellular movement, connective tissue development and function,’ the most representative IPA Network, with 35 proteins differentially expressed in FM fibroblasts. The different intensities of red and green represent overexpression or underexpression state of theses protein in FM fibroblast. Yellow highlighted proteins are those participating in ‘Connective tissue disorders.’

Furthermore, to better characterize how differentially expressed proteins are linked, an IPA *Network* tool analysis was conducted. According to this software, 35 proteins were related to ‘Cell morphology, cellular movement, connective tissue development and function,’ with a huge number participating in ‘Connective tissue disorders’ (Figure 3).

Additionally, those proteins differentially expressed in FM fibroblast were analysed with a different database, KEGG, to get further insight into their molecular roles. The five routes with a higher number of annotated proteins are shown in Table 3.

#### 3.1.3. Validations of Collagen Type 1 and 6 alpha 1 Chains and Fibronectin 1

**Figure 4:**
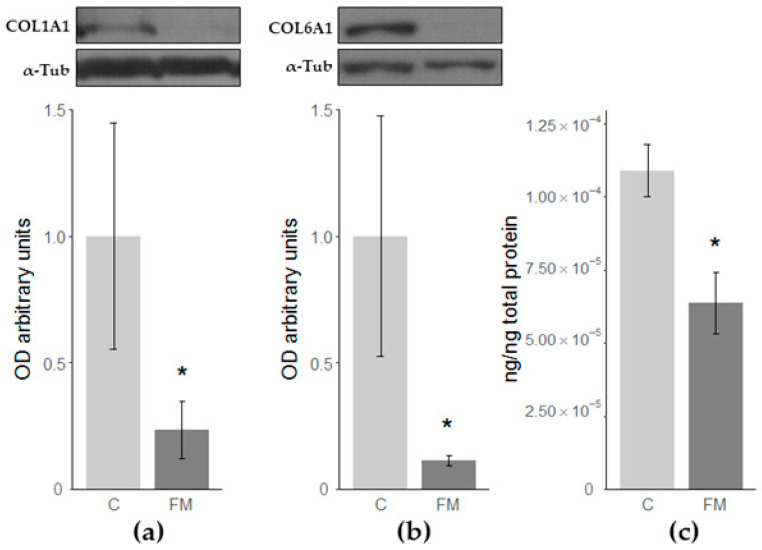
Densitometric quantifications of western-blot of (**a**) collagen type I alpha 1 chain (COL1A1) and (**b**) collagen type 6 alpha 1 chain (COL6A1) relative to α-tubulin (α-Tub). A representative immunoblot from a single experiment is shown; (**c**) Relative concentration of fibronectin 1 (FN1) determined by ELISA. Differences when comparing against C cultures: * *p*-value < 0.05.

Among the proteins differentially expressed in FM fibroblasts, collagen type 1 and 6 alpha 1 chains and fibronectin 1 showed drastic changes. Therefore, the differential expression of these proteins was confirmed by an alternative technique. As shown in Figure 4, western-blot and ELISA analyses confirmed the FM-associated statistically significant decrease in these proteins (Figure 4).

### 3.2. Effect of HT on Cell Proteome

To analyse HT effect on cells proteome, the fibroblasts from the patient were treated for 48 h with a nutraceutical relevant dose of HT (1.5 µM), and then analysed by nLC-MS/MS. Establishing a threshold in a substantial FC between C and FM cultures (FC < 0.5 or > 1.5 in FM/C), 91 differentially expressed proteins were reverted by HT. The list of reverted proteins is available in the supplementary material (Table S3).

#### 3.2.1. IPA Analysis

**Figure 5:**
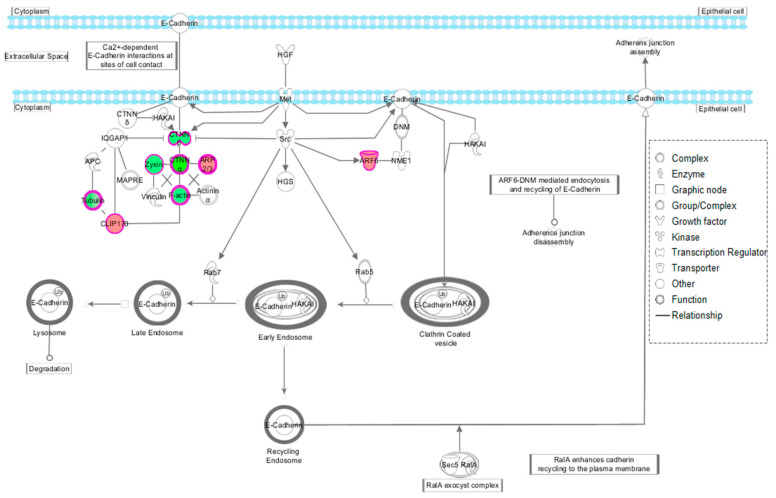
Schematic representation in IPA of the ¨Remodelling of epithelial adherens junction’ pathway.’ Overexpressed (red) and underexpressed (green) proteins in FM and normalized after HT-treatment.

Using the *Canonical Pathway* tool, it was possible to determine the pathways in which the HT-reverted proteins were involved. According to our results, the canonical pathway with the greatest statistical significance was ‘Remodeling of epithelial adherens junctions,’ with nine indexed proteins reverted by the treatment with HT (ACTB, ARF6, ARPC1B, CLIP1, CTNNA1, CTNNB1, TUBB6, TUBB and ZYX). Most of these proteins were key participants in cell-to-cell interactions through E-Cadherin (Figure 5).

**Figure 6:**
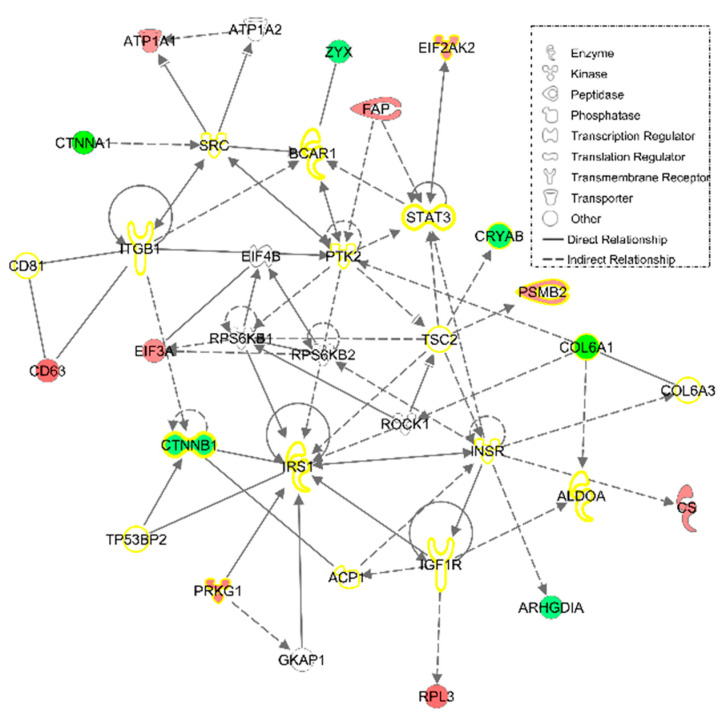
‘Cell death and survival, cellular movement, connective tissue development and function’ IPA network, the most representative one, with 15 out of 91 HT reverted protein in FM fibroblasts. The different intensities of red and green represent overexpression or underexpression state of theses protein in FM fibroblast before HT treatment. Empty nodes are proteins not found among the 91 HT-reverted proteins but used by IPA to build this network. Yellow-highlighted proteins are those participating in ‘Connective Tissue disorders.’

Additionally, an IPA *Network* tool analysis was conducted on these 91 proteins to know their degree of relationship, as well as their link with some physiological and pathological functions. Retrieving 15 out of 91 HT responsive proteins, the most relevant network was linked to ‘Cell death and survival, cellular movement, connective tissue development and function’ (Figure 6). Furthermore, HT treatment showed a normalizing effect on several proteins (yellow highlighted in the figure) participating in ‘Connective tissue disorders’ that have been pointed as differentially expressed in FM.

Finally, HT-reverted proteins were analyzed with an alternative database (KEGG) to investigate their role in cell metabolism. The five most representative routes (those with a higher number of annotated proteins) are listed in Table 4.

#### 3.2.2. Validations of the Reversion of Cofilin 1 and Catenin Beta 1 by HT

The effect of HT on the expression of cofilin 1 and catenin beta was confirmed by ELISA, since they were the two proteins with higher response and also involved in ‘ILK signalling’. However, we could only confirm the reversion of catenin beta 1 (Table 5).

## 4. Discussions

In the present work, we carried out, for the first time, an nLC-MS/MS study in primary dermal fibroblasts from a healthy volunteer and from a FM patient. Although this study was a case report, it aimed to be a first approach for future studies with more patients. Our results unveiled that fibroblast from FM showed a differential expression in proteins involved in the turnover of extracellular matrix (ECM) and oxidative metabolism that could explain the inflammatory status of these patients. Besides, a number of these proteins were normalized by the treatment with HT, which seems to support that an HT-enriched diet could be highly beneficial in the management of FM.

Among the 816 detected protein in both cultures, 357 showed differential expression in the fibroblast from the patient, composing a proteomic signature of FM. Of these proteins, 154 were upregulated and 203 downregulated in the culture from the patient. The PANTHER GO-Slim analysis of these proteins pointed to several processes linked to cytoskeleton arrangement (‘actin filament depolymerisation’), muscle-skeletal system development (‘connective tissue’, ‘cartilage’ and ‘bone development’) and mostly cell metabolism (‘ribosomal small subunit biogenesis’, ‘translation’ and their associated processes, ‘peptides’ or ‘amides biosynthetic processes,’ etc.). Among them, ‘actin filament depolymerisation’ was the term with the highest Fold Enrichment and the lowest FDR. Three proteins, overexpressed in the fibroblasts from the FM patient were annotated in this process: Alfa-adducin (ADDA), and alpha-1 and alpha-2 subunits of the F-actin capping protein (CAZA1 and CAZA2). Whereas ADDA attaches to actin filament extremes to protect them from depolymerisation [43], CAZA 1 and CAZA 2 proteins belong to the capZ protein complex that facilitates their assembly [44]. The homeostasis of actin filament has a crucial role in organelle distribution and dynamics. Indeed, actin depolymerisation participates in mitochondrial fission [45], a process whose impairment is linked to metabolic stress conditions [46]. Besides, the KEGG analysis showed that up to 115 FM-affected proteins participated in metabolic pathways. Hence, both results could explain, to some extent, those metabolic alterations described in FM [47,48,49]. In this line, ‘fatty acid β-oxidation’ and ‘tricarboxylic acids cycle’ were two mitochondrial metabolic routes activated in FM fibroblast, both of them with a significant z-score. The fibroblasts from FM patient showed four proteins overexpressed in this route: Alpha (ECHA) and beta (ECHB) subunits from hydroxyacyl-CoA dehydrogenase trifunctional multienzyme complex, hydroxyacyl-CoA dehydrogenase (HCDH) and acetyl-CoA acyltransferase 2 (THIM). The trifunctional multienzyme complex catalyses the last three steps in fatty acid β-oxidation [50]. When processing long chain fatty acids, enoil-CoA hydratase and 3-hidroxyacil-CoA deshydrogenase activities are developed by the alpha and beta subunits of this complex [51]. However, in short-chain fatty acid metabolism, these activities are managed by HCDH and THIM, respectively [52]. Acetyl-coA molecules produced in β-oxidation are further catabolized in the tricarboxylic acid cycle. In this regard, four proteins of this cycle were overexpressed in the fibroblasts from the FM patient: Citrate synthase (CISY), malate dehydrogenase 2 (MDHM), oxoglutarate dehydrogenase (ODO1) and dihydrolipoamide S-succinyltransferase (ODO2). The imbalance in these two relevant mitochondrial routes is indicative of an altered energy metabolism in FM. According to PANTHER analysis, some other key processes related with the metabolism of cells were also affected in the fibroblast from the FM patient: transfer RNA processing, protein translation and vesicle-associated transport from endoplasmic reticulum (ER) to Golgi apparatus (‘COPII-coated vesicle budding’). All these processes are somehow linked to protein processing, since once they are assembled in ER, proteins are glycosylated in the Golgi apparatus prior to being allocated to cell membrane, lysosomes or even being secreted. As a whole, these results unveil that protein synthesis and post-translational processing could be abnormal in FM.

Muscle-skeletal system also appeared to have some disrupted processes in the fibroblasts from the FM patient according to PANTHER analysis. ‘Skeletal system development’ and ‘bone morphogenesis’ are essential for a proper and fully functional muscle-skeletal system. Both terms are narrowly related with chondrocyte differentiation, cartilage-forming cells that are required in maintenance of bone-muscle junctions. Most of the proteins participating in these three routes are collagens: Alpha-1 and -2 chains of collagen type I (CO1A1 and CO1A2), alpha-1, -2 and -3 chains of collagen type VI (CO6A2, CO6A3 and CO6A1), and alpha-1 chain of collagen type XII (COCA1). All of them showed lower expression in fibroblasts from the FM patient when compared with those from the healthy volunteer. Type I collagen is ubiquitously present in human body, being the main component of organic mass from bones (up to 90%) and responsible for connective tissue formation and integrity [53]. In this line, when type I collagen synthesis is affected, the development of muscle-skeletal pathologies such as osteogenesis imperfecta is common [54]. The degradation of this collagen by matrix metalloproteinase 14 (MMP14) is crucial for ECM turnover [55]. This metalloproteinase showed a higher expression in the fibroblasts from the FM patient, a situation that could worsen collagen deficiency in FM. An altered ECM dynamic is related with acute and chronic inflammatory processes such as arthritis [56,57], a chronic disease with a large number of symptoms shared with FM. Inflammation is a key process in the response to endogenous/exogenous threats in which crosstalk among the immune system, coagulation pathway and the nervous system is essential. This response is managed by inflammatory mediators that can be classified as plasma-derived and cell-derived mediators [58]. In this context, there is evidence that collagen type I metabolites, together with those of elastin, activate the production of pro-inflammatory cytokines, contributing to the inflammatory situation [59,60]. In fact, the KEGG analysis on differentially expressed protein showed that 39 of those proteins participated in necroptosis, an hybrid type of cell death linked to inflammation [61], reinforcing its role in FM. On the other hand, it is known that collagen type I is associated with collagen type VI, a widely distributed collagen that showed lower expression in the fibroblasts from the FM patient. Type VI collagen is the main responsible for preserving the integrity of cartilage and bone, and its deficiency is related with several muscle-skeletal pathologies such as osteoarthritis, Bethelm and Ulrich myopathies, and cartilage-associated affections like Ehlers Danlos-like syndromes [62,63,64,65]. This last syndrome is considered a rare disease that affects skin, joints and blood vessels [66], and shares some symptoms like joint pain, anxiety, depression and chronic fatigue with FM. In fact, it was proposed that FM could be a nonclassified variant of hypermobile Ehlers-Danlos syndrome [67]. Furthermore, some mutations in the gene-codifying type XII collagen seem to be responsible for the lower expression of this protein and have also been linked to the development not only of Ehlers-Danlos syndrome but also of a Ulrich-like myopathy related with an impairment in ECM [68,69]. In fact, ECM-cell interactions seem to be altered in FM, since KEGG analysis showed up to 33 proteins participating in focal adhesion among the differentially expressed proteins in FM. Precisely, an adequate integrity of this structure relays on a proper ECM-cell cytoskeleton interaction, a highly specified junction in which integrins have a key role. These proteins and their associated kinases (ILK) participate in signalling processes between ECM and cells in a bidirectional manner. On the one hand, joining of talin to integrins allow their anchoring to ECM through collagen or fibronectin [70], all of them with lower expression levels in the fibroblast from the FM patient. On the other hand, ILK can rearrange actin filaments, managing several cell functions such as proliferation or motility [71,72,73]. According to IPA analysis, ILK signalling had a significant lower value of z-score (- 2.14) in fibroblasts obtained from the patient, reflecting the differences in this molecular cascade between the healthy volunteer and the FM patient. Thus, 12 (ACTB, COF1, COF2, CTNB1, FINC, FLNC, MYH9, MYL6, MYL9, MYPT1, NACAM and PARVA) out of 16 proteins annotated in this route had lower levels in FM culture when compared with healthy volunteer one. This finding, added to the previous finding about collagens levels confirmed by western blot, once again indicates the presence of alterations in the cytoskeleton as ECM-cell interactions in FM fibroblasts.

GP6 signalling was the last process which was pointed by IPA analysis as inhibited in FM fibroblasts. The glycoprotein VI is a transmembrane collagen receptor exclusively expressed in platelets that facilitates their aggregation in blood coagulation [74,75]. Thus, taking into account that our model is a dermal fibroblasts culture, this result has no biological relevance at all, although it could be explained by the drastic fall in the levels of collagen. In this line, this alteration is a key process to be addressed in the future in the platelets of our patient, given that we already observed an abnormal coagulation cascade in FM patients [30].

Once alterations of the energy metabolism and of ECM turnover were confirmed in fibroblasts from FM patient, we proceeded to test the possible modulatory effect of the phenolic compound HT on the proteome of these cells. On the one hand, the plasma concentration of HT after the intake of EVOO was highly variable depending of several factors such as the volume of intake or the olive cultivar. However, it can be assumed that, in humans, peak plasma concentrations of HT after the ingestion of 25 mL of EVOO can be around 0.1 µM [76]. On the other hand, HT is a compound of great interest for the nutraceutical industry due to its nonmutagenic and nongenotoxic profile of HT [77,78]. It has been described that the plasma concentration of HT reaches values of 2.83 µg/mL (around 18 µM) after the daily intake of two gastroresistant capsules (15 mg of HT per unit) for three weeks [79]. With this background, in this study, we decided to evaluate the effect of a dose in between those mentioned above (1.5 µM), which was relevant from a nutraceutical point of view and previously used in the literature [34,35,36]. After the nLC-MS/MS analysis of fibroblasts, we focused on those proteins from FM fibroblasts that, after HT treatment (FM+HT), showed similar levels to those expressed in fibroblasts from healthy volunteer (C). That is, those proteins that showed statistically significant differences in the FM/C comparison but not when comparing FM+HT *versus* C. With this criterion, 91 out of 357 proteins differentially expressed in fibroblast from FM patient were normalized with HT treatment and further analysed through IPA.

The most significant route normalized by HT was ‘remodelling of epithelial adherens junction’. Nine proteins participating in this route (ACTB, ARF6, ARPC1B, CLIP1, CTNNA1, CTNNB1, TUBB6, TUBB and ZYX) responded to HT treatment, and CTNNB1 was even confirmed by ELISA. Although these cell-to-cell junctions are typically related with epithelial cells, they can also be found among fibroblasts, especially in those tissues forming syncytia like the fibrous connective tissue [80]. In fact, IPA *Network tool* confirmed that most of the proteins affected by HT treatment were related with connective tissue, given that ‘connective tissue development and function’ was one of the terms used to define the most relevant network. Additionally, epithelial adherens junctions are also essential structures in the maintenance of the integrity of intestinal epithelial barrier, whose disruption are closely related with inflammatory pathologies such as Crohn disease or even FM [81,82]. Even though the phenolic compound curcumin has been able to protect the integrity of this cell-to-cell junction [83], little is known about the effect of phenolic compounds from EVOO on epithelial adherens junctions in humans. However, a study on a murine model revealed the ability of HT to repair the disrupted intestinal barriers [84]. Hence, further research is needed to ascertain whether the restorative effect of HT on the intestinal barrier could be linked to its effect on epithelial adherens junctions, and how it could help to ameliorate the proinflammatory component of FM and other inflammatory pathologies.

Besides, the 91 proteins normalized by HT were analysed by KEEG database. By this approach, we found that these proteins were also involved in: (i) Metabolic routes, (ii) ER protein processing, (iii) actin cytoskeleton regulation, (iv) thermogenesis and (v) proteoglycans, all of them involved one way or another in processes that were previously mentioned in FM/C comparison. Among the molecules participating in metabolic routes, we found proteins involved in some relevant processes, such as oxidative phosphorylation (f –ATPK– and B1 –AT5F1– subunits from ATP synthase F_0_ complex), tricarboxylic acid cycle (citrate synthase –CISY–) or carbohydrates (glutamine fructose-6-phosphate transaminase –GFPT1–; 1,4-alpha-glucan branching enzyme –GLGB–; glucose 6-phosphate isomerase –G6PI–) and lipids metabolisms (Very long chain 3-oxoacyl-coA reductase –DHB12–). Besides, additional processes, like heme group metabolism (Biliverdin reductase A –BIEA–), nucleotide synthesis (Tetrahydrofolate synthase C1 –C1TC–; Adenilosuccinate synthetase –PURA2–; transaldolase –TALDO–) or protein maturation (Catalytic subunit from Oligosaccharyltransferase enzymatic complex –STT3B–), were similarly responsive to HT treatment. This widespread effect of HT on different metabolic routes is a proof of its ability to restore the previously mentioned metabolic alterations found in FM. In fact, some of these normalizing effects on cell metabolism have been demonstrated in murine models [85] and human endothelial cells [86].

HT treatment also normalized the ER protein processing, affecting up to eight proteins (CRYAB, DNAJA2, ERO1A, E2AK2, SC23A, SC31A, STT3B and SAR1A). This organelle is directly responsible for the synthesis, assembly and maturation of more than a third part of all the proteins in the cell [87]. When unfolded proteins are accumulated within the ER, a molecular cascade is activated in order to counteract the deleterious effect linked to this alteration, worsen by inflammatory processes [88]. The regulatory effect exerted by HT on this organelle was previously tested on human hepatocarcinoma cells [89] and animal models [90]. Since not only ER but also the vesicle-associated transport by COPII between the ER and Golgi apparatus seem to be affected in FM, the regulatory effect exerted by HT on organelle could be beneficious in ameliorating protein processing within the cells. As well as the ER and its associated transport, actin cytoskeleton was also regulated in FM fibroblasts under HT treatment. Concretely, five proteins (ACTB, ARC1B, COF1, COF2 and MYPT1) were normalized, a quite interesting result taking into account that this structure was one of the most affected in the FM/C comparison. To our knowledge, HT effect on actin cytoskeleton has not been studied until now, with scarce studies regarding how other phenolic compound could alter its conformation [91]. However, actin cytoskeleton plays a crucial role on cell anchoring to ECM, and HT could regulate ECM dynamics by inhibiting its remodelling processes [92]. In this regard, HT exerted a direct effect on several proteins involved in ‘Cell death and survival, cellular movement, connective tissue development and function’ and, even more, in those related to ‘Connective tissue disorders’ like catenin β (CTNB1), confirmed by ELISA. Besides, HT treatment normalized the levels of five proteins (ACTB, CD63, CTNB1, MYPT1 and KAPCA) related with proteoglycans pathway in the fibroblasts from FM patient. Proteoglycans are cell surface/ECM macromolecules, composed by one or more glycosaminoglycans covalently joined to a membrane or secreted protein [52]. These molecules are closely related with muscle-skeletal system pathologies such as Schwartz-Jampel syndrome or osteochondritis dissecans, whose patients suffer from muscle weakness or joint problems [93,94]. Even more, proteoglycans have been proposed as serum biomarkers for several inflammatory-related syndromes like different types of arthritis or FM [95,96]. Altogether, these results about the effect of HT on ECM and proteoglycans reinforce the point to this potential treatment in FM. Nonetheless, it would be necessary further investigation to assess the particular effect of HT on ECM metabolism and its link to their anti-inflammatory properties.

The KEGG analysis also unveiled the effect of HT on thermogenesis pathway and its normalizing effect on five proteins of this route (ACTB, ATPK, AT5F1, KAPCA and KGP1). It has been demonstrated that thermoregulation is an inefficient process in FM patients [97]. As a result, these patients have an abnormal low body temperature, especially around trigger points [98]. This points are distributed through the body following a very similar pattern to that followed by brown adipose tissue deposits [99] and, curiously, thermogenesis-activating stimuli such as stress and cold also worsen FM symptoms [100,101]. Some authors have pointed that a disruption in ECM-adipocyte receptors interaction could be responsible for an impaired thermogenesis [102]. According to our *in vitro* model, the interaction between ECM and cell receptors were compromised in FM fibroblasts and, besides, the mitochondrial metabolism was impaired in these cells. Taking together the effect that the HT exert on these processes could somehow improve some of the alterations found in FM.

## 5. Conclusions

In conclusion, the comparative proteomic analysis of fibroblasts cultured from a healthy woman and a patient of FM indicates the alteration in the homeostasis of the ECM, cell cytoskeleton and energy metabolism, as well as their possible role in the proinflammatory state associated with the pathology. Interestingly, these alterations could be reversed, at least partially, by a nutraceutical relevant dose of HT, supporting the usage of an HT-enriched diet as a beneficial approach in the management of FM. Further studies with more doses, more patients, and with hydroxytyrosol sulphate, the main metabolite of this simple phenol in human plasma, could be performed to gain insight into the effect of HT in this pathological situation.

## Figures and Tables

**Table 1:** PANTHER GO-Slim output list of Biological Processes related with differentially expressed proteins in the fibroblasts from the FM patient.

PANTHER GO-Slim Biological Process	GO ID	Fold Enrichment	FDR
actin filament depolymerisation	GO:0030042	15.97	5.04 × 10^−2^
COPII-coated vesicle budding	GO:0090114	15.97	4.95 × 10^−2^
bone morphogenesis	GO:0060349	13.78	2.02 × 10^−2^
chondrocyte differentiation	GO:0002062	11.71	2.74 × 10^−2^
connective tissue development	GO:0061448	11.16	2.86 × 10^−2^
cartilage development	GO:0051216	11.16	2.80 × 10^−2^
bone development	GO:0060348	10.65	3.10 × 10^−2^
cellular protein complex disassembly	GO:0043624	8.57	9.17 × 10^−3^
ribosomal small subunit biogenesis	GO:0042274	7.74	5.72 × 10^−3^
translational initiation	GO:0006413	7.51	3.03 × 10^−2^
protein-containing complex disassembly	GO:0032984	7.10	3.29 × 10^−3^
cytoplasmic translation	GO:0002181	6.76	2.15 × 10^−2^
cellular amino acid metabolic process	GO:0006520	6.71	4.41 × 10^−6^
translational elongation	GO:0006414	6.25	4.29 × 10^−9^
translation	GO:0006412	6.25	3.22 × 10^−9^
peptide biosynthetic process	GO:0043043	6.17	2.77 × 10^−9^
amide biosynthetic process	GO:0043604	5.88	2.93 × 10^−9^
ribonucleoprotein complex assembly	GO:0022618	5.70	1.03 × 10^−3^
carboxylic acid catabolic process	GO:0046395	5.54	2.14 × 10^−2^
organic acid catabolic process	GO:0016054	5.54	2.09 × 10^−2^

**Table 2:** Proteins listed in the four canonical pathways with statistically significant z-score.

Symbol	Entrez Gene Name	UniProt Accession	q-Value	Fold Change	Pathway
PTPA	protein phosphatase 2 phosphatase activator	PTPA_HUMAN	3.82 × 10^−5^	1.44	ILK
VIM	vimentin	VIME_HUMAN	1.16 × 10^−4^	1.40	
ITGB1	integrin subunit beta 1	ITB1_HUMAN	3.26 × 10^−3^	1.38	
CDC42	cell division cycle 42	CDC42_HUMAN	5.28 × 10^−3^	1.22	
MYH9	myosin heavy chain 9	MYH9_HUMAN	2.27 × 10^−3^	−1.31	
MYL6	myosin light chain 6	MYL6_HUMAN	2.05 × 10^−2^	−1.31	
NACA	nascent polypeptide associated complex subunit alpha	NACAM_HUMAN	1.00 × 10^−3^	−1.47	
PARVA	parvin alpha	PARVA_HUMAN	4.29 × 10^−4^	−1.47	
CFL1	cofilin 1	COF1_HUMAN	3.53 × 10^−3^	−1.50	
ACTB	actin beta	ACTB_HUMAN	3.82 × 10^−5^	−1.52	
CTNNB1	catenin beta 1	CTNB1_HUMAN	5.75 × 10^−4^	−1.64	
MYL9	myosin light chain 9	MYL9_HUMAN	3.16 × 10^−4^	−1.73	
CFL2	cofilin 2	COF2_HUMAN	1.32 × 10^−3^	−1.73	
PPP1R12A	protein phosphatase 1 regulatory subunit 12A	MYPT1_HUMAN	1.04 × 10^−3^	−1.86	
FLNC	filamin C	FLNC_HUMAN	4.22 × 10^−4^	−1.95	
FN1	fibronectin 1	FINC_HUMAN	1.62 × 10^−5^	−3.31	
ACAA2	acetyl-CoA acyltransferase 2	THIM_HUMAN	3.67 × 10^−4^	1.82	βOX
HADH	hydroxyacyl-CoA dehydrogenase	HCDH_HUMAN	1.80 × 10^−2^	1.82	
HADHB	hydroxyacyl-CoA dehydrogenase trifunctional multienzyme complex subunit beta	ECHB_HUMAN	2.57 × 10^−4^	1.49	
HADHA	hydroxyacyl-CoA dehydrogenase trifunctional multienzyme complex subunit alpha	ECHA_HUMAN	2.46 × 10^−2^	1.48	
CS	citrate synthase	CISY_HUMAN	7.93 × 10^−3^	1.72	TCA
MDH2	malate dehydrogenase 2	MDHM_HUMAN	4.67 × 10^−3^	1.57	
OGDH	oxoglutarate dehydrogenase	ODO1_HUMAN	6.99 × 10^−3^	1.53	
DLST	dihydrolipoamide S-succinyltransferase	ODO2_HUMAN	1.08 × 10^−3^	1.21	
RAC1	Rac family small GTPase 1	RAC1_HUMAN	1.06 × 10^−2^	1.35	GP6
COL6A2	collagen type VI alpha 2 chain	CO6A2_HUMAN	1.07 × 10^−3^	−1.22	
COL6A3	collagen type VI alpha 3 chain	CO6A3_HUMAN	2.43 × 10^−2^	−1.40	
COL6A1	collagen type VI alpha 1 chain	CO6A1_HUMAN	1.60 × 10^−4^	−2.76	
TLN1	talin 1	TLN1_HUMAN	3.00 × 10^−4^	−3.70	
COL12A1	collagen type XII alpha 1 chain	COCA1_HUMAN	1.04 × 10^−4^	−4.90	
COL1A2	collagen type I alpha 2 chain	CO1A2_HUMAN	1.34 × 10^−5^	−7.38	
COL1A1	collagen type I alpha 1 chain	CO1A1_HUMAN	1.49 × 10^−3^	−11.06	

ILK: Integrin-linked kinase signalling; βOX: Fatty acid β oxidation; TCA: Tricarboxylic acid cycle; GP6: Glycoprotein VI signalling pathway.

**Table 3:** KEGG analysis of proteins with differential expression in FM.

KEGG ID	Name	N° of Proteins
hsa01100	Metabolic pathways	115
hsa05200	Pathways in cancer	44
hsa04217	Necroptosis	39
hsa05165	Human papillomavirus infection	34
hsa04510	Focal adhesion	33

For protein and KO identifier annotation in each pathway, please consult Supplementary Table S2.

**Table 4:** KEGG analysis of the ninety-one proteins reverted by HT.

KEGG ID	Name	N° of Proteins	Proteins Abbreviation
hsa01100	Metabolic pathways	12	ATPK, AT5F1, BIEA, CISY, C1TC, DHB12, GFPT1, GLGB, G6PI, PURA2, STT3B, TALDO
hsa04141	Protein processing in endoplasmic reticulum	8	CRYAB, DNAJ2, SC23A, SC31A, STT3B, ERO1A, E2AK2, SAR1A
hsa04810	Regulation of actin cytoskeleton	5	ACTB, ARC1B, COF1, COF2, MYPT1
hsa04714	Thermogenesis	5	ACTB, ATPK, AT5F1, KAPCA, KGP1
hsa05205	Proteoglycans in cancer	5	ACTB, CD63, CTNB1, MYPT1, KAPCA

For protein abbreviation interpretation, please consult Supplementary Table S3.

**Table 5:** Concentration (ng/ng total protein) of COF1 and CTNB1, two HT-reverted proteins validated by ELISA. Data are represented as average ± SD.

Symbol	CONTROL	FM	FM+HT
COF1	1,41 × 10^−7^ ± 6,69 × 10^−9^	2,24 × 10^−7^ ± 1,73 × 10^−9^ ***	2,10 × 10^−7^ ± 8,46 × 10^−10^ ***
CTNB1	7,15 × 10^−2^ ± 2,45 × 10^−3^	5,11 × 10^−2^ ± 1,21 × 10^−3^ **	6,64 × 10^−2^ ± 1,205 × 10^−3^

Differences when compared against CONTROL cells: ** *p*-value < 0.01; *** *p* < 0.001.

## Supplementary Materials

The following are available online at https://www.mdpi.com/2072-6643/12/8/2386/s1, Table S1: List of proteins differentially expressed between FM and C, Table S2: List of proteins and KO identifiers annotated in each KEGG pathway linked to those proteins differentially expressed between FM and C. Table S3: List of proteins reverted by HT treatment in the fibroblasts culture from FM patient.
